# 

**Published:** 1874-10

**Authors:** 


### CONTENTS.

PAGE.

**CONTRIBUTIONS.**

Profit and Loss. BEN. H. PRATT, A. M. ............... 275
Affectation in Chronic Invalidism. P. H. HAYES, M.D. ............... 277
Patent Medicines. M. S. BIDWELL ............... 279

**MEDICAL ITEMS AND NEWS.**

Antidote for Phenic Acid—Gelseminum in Rheumatism—Incontinence of Urine in Old People—Diabetes—Headache after Drunkenness—Iodine Caustic—Pneumonia—Freckles—Agreeable Purgative—Diphtheria—Freckles Again—Compound Syrup of Garlic—Toothache Drops—Caseous Pneumonia—Hæmorrhoids—Syphilis—Cerebro-Spinal Meningitis—Puerperal Convulsions—Jalap Biscuits—Bichloride of Methylene—Menorrhagia—Pain in Bowels—Chloral as Anæsthetic during Labor—Fetid Feet—Cholera Morbus—Diphtheria—Uterine Neuralgia—Sciatica—Rheumatic Liniment—Acetate of Lead Lotion—Balsamic Cigarettes for Asthma—Hair Tonic—To Administer Creosote—Intermittent Fever—Liniment—Diaphoretic—Elixir Chloroform—Chronic Bronchitis—Dysentery—Liquor Sedativus—Acute and Subacute Rheumatism—Prurigo and Pruritus—To Deprive Iodine of Stain—To check Vomiting from Cough—Irritable Heart—Facial Neuralgia—Tamarind Cough Mixture—Seborrhœa—Syrup of Borax—Tape Worms—Emulsion of Raw Meat—Quarana in Chronic Rheumatism—Carbuncle treated by Carbolic-acid—Cod Liver Oil—Nicotine an Antidote to Strychnia ............... 280 to 287

**OUR CORRESPONDENCE.**

Nine Letters with Replies ............... 287

PAGE.

**HOUSEHOLD HINTS AND RECIPES.**

To Bleach Sponges—Cement for Aquaria—Preservation of Essence of Lemon—Mushroom Catsup—Alcohol in various "Bitters"—Substitute for Milk or Cream—Corns—To Remove Iron-Rust—Salad Dressing—Soft Cheese—Crimson Marking Ink—Hair Curling—Walnut Catsup—Shaving Fluid—Mock Venison of Corn Beef—Breakfast Dish—Indelible Red Ink—Pumpkin Preserves—Grease, Tar, Pitch and Paint—Boudet's Depilatory—Stove Blacking—Simple Remedy for Dandruff—Coloring Wash for Fences—Breakfast Dish—To Remove Iron taste from new Kettles—Chicken Cheese—Trap for Insects—Pickling Green Corn—Tanning Leather—Coffee and Milk—Fat Meat for Consumptives—Why a Child Loves Sugar—New Method of making Beef Tea—To restore Scratched Furniture ............... 288 to 293

**EDITORIAL.**

That Law ............... 293

Knock-Knee ............... 293

Near and Long Sightedness ............... 294

Reading after Confinement ............... 296

**CHIT-CHAT.**

The County Fair—Wanted—Read It—New Physician—Old Subjects—Don't—Blessing After All—Olden Time Surgery—Swallowing a Tool-Chest—A Granger—Quack Medicine—Inconvenience of Deafness—The Meat Question—The Liquor Dealers—Why Not?—That Infernal Noise—From H.—Splendid Organ—Didn't Pay—Dental Bill—Japanese Medical Journal—Annoyance—Just Think of It!—Needs Protection—Try It—Dusting—Tapeworm in Grouse—Our Barometer—Whew! ............... 297 to 302

BOOKS AND PERIODICALS ............... 302

